# Surface Engineered Iron Oxide Nanoparticles Generated by Inert Gas Condensation for Biomedical Applications

**DOI:** 10.3390/bioengineering8030038

**Published:** 2021-03-15

**Authors:** Aver Hemben, Iva Chianella, Glenn John Thomas Leighton

**Affiliations:** Surface Engineering and Precision Institute, Cranfield University, Bedfordshire MK430AL, UK; hemben@cranfield.ac.uk (A.H.); i.chianella.1998@cranfield.ac.uk (I.C.)

**Keywords:** iron oxide nanoparticles, PVD, Mantis NanoGen Trio, drug delivery

## Abstract

Despite the lifesaving medical discoveries of the last century, there is still an urgent need to improve the curative rate and reduce mortality in many fatal diseases such as cancer. One of the main requirements is to find new ways to deliver therapeutics/drugs more efficiently and only to affected tissues/organs. An exciting new technology is nanomaterials which are being widely investigated as potential nanocarriers to achieve localized drug delivery that would improve therapy and reduce adverse drug side effects. Among all the nanocarriers, iron oxide nanoparticles (IONPs) are one of the most promising as, thanks to their paramagnetic/superparamagnetic properties, they can be easily modified with chemical and biological functions and can be visualized inside the body by magnetic resonance imaging (MRI), while delivering the targeted therapy. Therefore, iron oxide nanoparticles were produced here with a novel method and their properties for potential applications in both diagnostics and therapeutics were investigated. The novel method involves production of free standing IONPs by inert gas condensation via the Mantis NanoGen Trio physical vapor deposition system. The IONPs were first sputtered and deposited on plasma cleaned, polyethylene glycol (PEG) coated silicon wafers. Surface modification of the cleaned wafer with PEG enabled deposition of free-standing IONPs, as once produced, the soft-landed IONPs were suspended by dissolution of the PEG layer in water. Transmission electron microscopic (TEM) characterization revealed free standing, iron oxide nanoparticles with size < 20 nm within a polymer matrix. The nanoparticles were analyzed also by Atomic Force Microscope (AFM), Dynamic Light Scattering (DLS) and NanoSight Nanoparticle Tacking Analysis (NTA). Therefore, our work confirms that inert gas condensation by the Mantis NanoGen Trio physical vapor deposition sputtering at room temperature can be successfully used as a scalable, reproducible process to prepare free-standing IONPs. The PEG- IONPs produced in this work do not require further purification and thanks to their tunable narrow size distribution have potential to be a powerful tool for biomedical applications.

## 1. Introduction

Over the last two decades, nanoparticles have captured much research interest due to their great potential for biomedical applications. Due to the need for improved diagnostics and therapeutics methods for musculoskeletal ailments, scientists have been exploring the use of several types of nanomaterials, including metal nanoparticles conjugated with biological molecules both for diagnostics and for delivery of drugs only to affected organs/tissues reducing adverse side effects and improving therapy [1]. Physical, chemical and biological methods for developing metal nanoparticles include mechanical milling, physical vapor deposition and sputtering [2]. Chemical reactions develop nanoparticles via precipitation [3] of ions followed, in many cases, by their oxidation or reduction [4]. Other chemical methods available for the synthesis of metal nanoparticle include sol gel [5], co-precipitation, particularly for iron oxide nanoparticles [6] colloidal [7] and hydrothermal nanoparticle synthesis [7,8], gas phase methods [8] and electrodeposition [9]. Further development has led to other methods including polymer matrix-mediated synthesis [10], precipitation using microemulsions [11] and vesicles [12].

Among all the metal nanoparticles, iron oxide nanoparticles (IONPs) are one of the most promising for biological applications [13,14]. In fact, they can be easily produced with magnetic properties (i.e., IONPs with uniform surface and sizes < 20 nm are often superparamagnetic [15]) that facilitate their handling during chemical/biological functionalization as well as visualization by magnetic resonance imaging (MRI) when inside the body for clinical diagnostics Thanks to their clinical safety, utility and versatility IONPs have actually already been used in medicine for decades. Due to their property to slowly release iron, iron oxide-cabohydrates complexes or colloids have been used for the treatment of iron deficiency anemia. Similarly, due to IONPs’ magnetic fluid hyperthermia, which is the property to produce heat locally when the nanoparticles are exposed to an alternating magnetic field, they have been exploited to kill cells in cancer tissues for an effective and safe (minimal side effects) cancer therapy [16]. IONPs display different magnetic characteristics according to the method of production as a result of structural disorder [17], creation of antiphase boundaries [18] or existence of mechanically dead layers at the surface of the particles [19]. Paramagnetic interaction between particles and flocculation due to Vander Waals forces necessitates surface modification in the form of a dense coating to achieve particle stability. In addition to magnetic forces between single particles, within the magnetic field, magnetic interactions between particles clusters can also take place [20].

According to [21] further research exploring fabrication techniques for nanomaterials, especially applications of IONPs with diverse features, will contribute to innovation in a variety of areas. Optimized and scalable synthesis is critical for the development of nanomaterials to be used in pre-clinical and clinical applications [22]. Other physical, chemical, thermal and mechanical characteristics of IONPs that can be exploited in biomedical applications include cellular labelling and separation, tissue repair, drug delivery, magnetic resonance imaging hyperthermia and magnetofection [20,21,22]. With the frequent investigation of the use of metal nanoparticles in biological systems, IONPs toxicity levels have been assessed in several scientific investigations. IONPs modified with biocompatible polymers exhibit low level toxicity, when used in diluted conditions [23]. They have also shown useful blood circulation time and cells’ internalization efficiency [23].

In order to improve their stability and biocompatibility, IONPs can be dispersed in specific inorganic or polymeric solutions [24] as well as in natural gelatin, dextran and chitosan solutions [24,25,26]. Without modification, IONPs possess hydrophobic surfaces and a large surface area to volume ratio. The hydrophobic intra particulate interactions cause the iron oxide particles to agglomerate and produce larger clusters with a resultant increase in particle size with changes in magnetic attractions [25]. Synthetic polymers used for coating IONPs include poly (ethylene-co-vinyl acetate), poly (vinylpyrrolidone), poly (lactic-co-glycolic acid), poly (ethylene glycol) (PEG), poly (vinyl alcohol) and polyethylene imine (PEI) [25,26]. In addition to increasing stability, coatings of PEG and short chain PEI have been shown to enhance biocompatibility [26]. PEG can be non-covalently immobilized on the nanoparticle surface to enhance their properties as nanocarriers for drug delivery. Intracellular delivery of drugs into specific tissues and organs for gene therapy remains a major challenge [26]. Nanoparticles, including IONPs, have the potential to facilitate gene delivery only to the affected tissues without or with minimal accumulation on healthy tissues.

Production of metal nanoparticles by inert gas condensation (IGC) and magnetron sputtering is a technique developed for generating and depositing nanoparticles on a substrate [27,28,29,30] from a target attached to a magnetron suspended in an inert gas- filled agglomeration chamber [31]. IGC and magnetron sputtering is recognized as a convenient and low costs method to produce metal nanoparticles on a substrate with desired composition, size, structure, shape and defect density [32]. Although the Mantis NanoGen Trio IGC system, utilized in this work, was used to generate and deposit ‘simple’ IONPs, the instrument has positions for potentially three different metal targets, enabling, if desired, sputtering and deposition of multi-metal nanoparticles [31]. IGC with magnetron sputtering has been used to sputter several types of metal nanoparticles including aluminum [33], copper [34], molybdenum [35], platinum [27], titanium [36] and iron oxide nanoparticles. Titanium oxide nanoparticles < 5 nm were obtained by IGC for applications in gas sensors and photo-catalytic processes [36]. Although IONPs are already known to be biocompatible [16,37,38], to the best of our knowledge, this is the first time that IGC has been used as a method for producing free standing PEG coated IONPs, which, therefore, are highly likely to be biocompatible and this will be confirmed in the future. Hence, this work describes for the first time the use of IGC for production of PEGylated, free-standing, soft landed IONPs with narrow size distribution with, therefore, great potential for application in drug delivery. In fact, the small size and narrow size distribution of the particles described in this work are an advantage over the comparatively wider size distribution produced by other methods [39]. Smaller nanoparticles are suitable for use in biomedical applications, especially when drugs need delivering inside cells. In fact, their small size not only permits the nanoparticles to cross the cells membrane, but allows for a more efficient excretion, hence minimizing accumulation in organs and tissues, where otherwise they may cause long term damage. One of the main features that affects biodistribution of nanomaterials in the body is their size and size distribution [40]. Therefore, it is very important to have tailored nanoparticles with narrow size distribution, as for such particles the biodistribution and excretion from the body can be predicted.

Hence, the work here demonstrates that by using the IGC via the Mantis NanoGen Trio system, IONPs with narrow size distribution, coated with biocompatible polymers such as PEG can be synthesized at ambient temperature in a reproducible way with minimal user intervention. Once synthesized the IONPs were characterized and their biocompatibility and use for drug delivery is currently under investigation.

## 2. Materials and Methods

### 2.1. Materials and Equipment

Phosphate buffered saline (PBS), RNAse free water and PEG 1000 Mwt were purchased from Sigma (Dorset, UK), while acetone was purchased from Fisher Scientific (Loughborough, UK). Iron oxide (Fe_3_O_4_) targets were purchased from Kurt J. Lesker Company Ltd. (East Sussex, UK). Mantis NanoGen Trio was purchased from Mantis Deposition Ltd. (Thame, UK) and was coupled to a Chemical Vapor Deposition chamber purchased from Scientific Vacuum Systems (Wokingham, UK).

### 2.2. Preparation of the Silicon Wafer Substrates

Silicon wafers (1 inch purchased from Agar Scientific Ltd, Essex, UK), were first plasma cleaned for 3 min in oxygen using a plasma cleaner purchased from Diener Plasma Surface Technology (Ebhsausen, Germany), to remove surface impurities and reduce hydrophobicity of the surface. PEG dissolved in acetone (0.2 g/mL) was then spin-coated on the clean wafers at 3000 rpm using a spin coater purchased from Electronic Micro Systems Ltd., (Sutton Coldfield, UK). In order to assess the volume of PEG spin coated on the surface, the wafers were weighed before and after spin coating with the difference in weight representing the mass of PEG attached. After modification with PEG, the wafers were characterized by contact angle measurements taken by a tensiometer equipped with Attension software, Biolin Scientific (Stockport, UK) and using deionized (DI) water. Measurement of the thickness of the PEG layer was conducted using DektakXT stylus profiler, Bruker (Coventry, UK).

### 2.3. Production of IONPs by Mantis NanoGen Trio System

The Mantis NanoGen Trio with three 1-inch diameter targets system sputters into the argon filled agglomeration chamber during formation of a plasma. During the 2-h process conducted here at ambient temperature (20 ºC), the target fixed on the top within chamber was used to generate IONPs in argon with a flow rate between 10–100 sccm. Production of IONPs using high or low power achieved by regulating the current applied to the magnetron involved very gradual increments of current (0.001 A per minute) due to the thermoplastic nature of the targets, while generating the plasma. The process was monitored at 30-min intervals. Pressure in main chamber was altered by changing the throttle position (0%–70%) and flow rate of argon (Scheme S1 in Supplementary information). Distance from the magnetron to the agglomeration chamber with the target was fixed at 13.8 cm. The IONPs produced in this work were pre-sputtered with the shutter closed for 15 min, to clean the target. The shutter was then opened and condensed IONPs, which have attained ground state, were channeled through the mass quadrupole filters to the PEG coated substrate surface. After deposition, PEG coated free- standing IONPs were obtained by immersing the 1-inch silicon wafer substrate in 15 mL RNAse free water in a glass beaker and ultrasonicating for 3 min to dissolve the PEG layer and disperse the IONPs in solution. The PEG added at the time of IONPs preparation prevents aggregation of the free-standing nanoparticles when these are dispersed in RNAse free water [41].

### 2.4. Characterization of Coated Silicon Wafers and Free Standing IONPs by AFM and TEM

Atomic force microscopy (AFM), performed using a microscope from Digital Instrument (Boston, USA), was used to characterize the PEG IONPs after production using a 2× magnification.

The PEG IONPs were also characterized by Transmission Electron Microscopy (TEM) using a JEM TEM Jeol instrument (Hertfordshire, UK). For both AFM and TEM measurements, aliquots of the PEG IONPs solutions were dropped either on a silicon wafer (for AFM) or on a TEM grid and dried in atmosphere before imaging. The TEM grid used had 3 mm diameter and a holey carbon support film.

### 2.5. Characterization of IONPs by Dynamic Light Scattering

Dynamic Light Scattering (DLS) measurements were conducted using a Nanosize instrument from Malvern Panalytical Ltd. (Malvern, UK) to assess the PEG IONPs size distribution and measure the amount of PEG surrounding the nanoparticles after production. To prepare the PEG IONPs samples, the solution with dispersed PEG IONPs, obtained after IGC production, was concentrated (3×) by evaporating the liquid at 50 °C for two hours in a centrifugal vacuum concentrator, Eppendorf 5301 from Sigma (Dorset, UK) and this was measured by DLS. In order to prepare a control containing only PEG, a silicon wafer was spin-coated with PEG (0.2 g/mL) and directly dissolved in 15 mL of RNAse free water with ultrasonication for 3 min. Then, similarly to the solution with the PEG IONPs, this was concentrated (3×) by the vacuum concentrator and measured by DLS. 

### 2.6. Characterization of IONPs by NanoSight Nanoparticle Tracking Analysis (NTA) 

A NanoSight LM20 from Malvern Panalytical Ltd (Worcester, UK) configured with a CCD camera and a red laser light was used to determine the size of the free-standing IONPs in RNAse free water (concentrated 3 times) and compared with the PEG in RNAse free water (also concentrated 3 times). For the measurements, 1 mL of 100 nm polystyrene spheres standard was injected into the NanoSight column to calibrate the instrument before loading each sample. The measurements were displayed by viewing the ’Thumbprint’ via NanoSight 3.4 version software connected to a monitor display. Next, 1 mL of each sample was injected into the instrument column and captured for 60 s at a constant temperature of 22 °C. Each sample was measured 3–5 time and the results were reported as averaged histograms.

## 3. Results

### 3.1. Silicon Wafer Substrate Characterization 

Silicon wafers are among the most common substrates for use in a pressurized main chamber. Preparation of the substrate for deposition of IONPs involved plasma cleaning to increase wettability of the substrate surface and enabling deposition of a thin PEG layer using a spin-coater. The polymer was applied to the wafer surface to enhance soft-landing and subsequent capture of the IONPs as well as to assure, after polymer dissolution, the achievement of free-standing PEG coated IONPs. Contact angle measurements using DI water conducted with a Tensiometer showed variation from bare, PEG coated and IONPs soft landed on PEG coated silicon wafer surface Figure S1 (Supplementary information).

Contact angle measurements on the bare, non-plasma cleaned wafer showed hydrophobicity with a contact angle of ~55°, while PEG – coated wafer showed a contact angle of ~7.5°. After sputtering IONPs on PEG-coated wafer, the layer showed a contact angle of ~14°. These results imply that the thin layer of PEG reduces the hydrophobicity of the silicon wafer as compared with the non-plasma cleaned bare surface. In addition, a small change on the wettability of the surface was also observed after sputtering the IONPs, which could be attributed to the presence of the particles on the PEG-coated wafer. The thickness of the PEG layer was determined along a scratch by using a DektakXT stylus profiler Figure S2 (in Supplementary information). The polymer thickness was determined at five points along the scratch resulting in an average 0.26 µm ± 0.031 µm. The amount of PEG spin coated on the silicon wafer was estimated by the difference in weight of the wafer between before and after spin-coating and it was found to be 15.8 mg ± 3.6%, for experiments performed in triplicates. This was considered a suitable amount of polymer to obtain IONPs deposition.

### 3.2. IONPs Production

IONPs with size between ~ 1–6 nm were produced using the Mantis NanoGen Trio system by application of power to the magnetron. The magnetron transmits energy to the target and to the pressurized inert gas in the agglomeration chamber. Energized supersaturated metallic vapor from the iron oxide target escape the surface of the target and interact with particles of the inert gas. As the process continues over time, the interplay of particulate collision and transfer of energy results in particles sputtering followed by their condensation and deposition onto a commercial 1-inch wafer. In the pressurized chamber supplied with power (20–49 Watts) and with argon as the inert gas carrier, regulation of the gas can result in variation of particle sizes. The instrument used in this work was coupled with a scientific vacuum systems (SVS) main chamber through a software (MesoQ), which also provided control over operating conditions such as chamber pressure, temperature, working distance (WD) and throttle position. The parameters for sputtering can be altered to achieve pre-determined nanoparticles size and size distribution. All the conditions tested in this work are summarized in Table S1 (in Supplementary Information).

**Figure 1:**
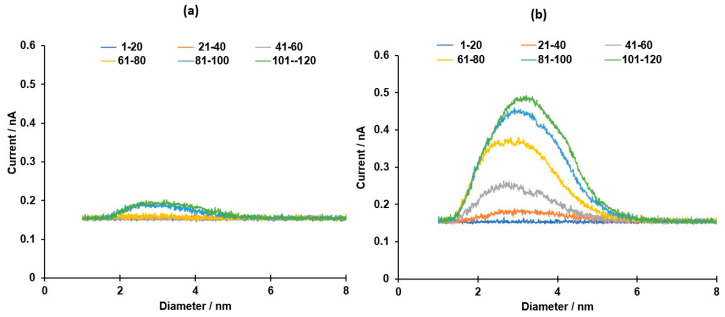
Low (**a**) and high (**b**) power spectra of Fe_3_O_4_.

Process parameters were adjusted to commence sputtering, while maintaining a stable plasma, Table S2 (Supplementary Information). In addition, the larger the WD between the magnetron head and the agglomeration chamber, the larger the size of IONPs produced [31] and in this work the WD was fixed to 13.8 cm, which in the optimized conditions produced particles with size between ~ 1–6 nm. During a 2 h sputtering session, the generation of nanoparticles in the chamber, shown as spectra of diameter vs. current, was recorded using the MesoQ controller software and exported to Excel. An average of 20 spectra was plotted during low (20 Watts) and high (49 Watts) intensity sputtering (Figure 1a,b).

Comparatively, the average spectra are for the purpose of data presentation and clarity, to depict the production of nanoparticles and show evolution. The spectra show intensity of sputtering as well as the size distribution of the nanoparticles generated in the agglomeration chamber. Prior to commencing IONPs sputtering and whilst the parameters are being optimized to achieve a stable plasma, the spectra are not distinguishable. When a stable plasma is achieved, however, spectra evolution can be seen and, in this work, after 15 min of sputtering with the shutter closed to clean the target, the shutter was opened to allow deposition of nanoparticles on the substrate. In the case of low power sputtering, the generation of the IONPs and the evolution of spectra were recorded for 2 h, while maintaining the minimum power (20 Watts or 531.9 V, 0.038 A) necessary to produce a stable plasma. These conditions produced spectra with low intensity particles generation (Figure 1a). For high power sputtering (49 Watts or 454.0 V, 110.0 A), however, the power was increased gradually after successfully striking a stable plasma until the maximum value recommended by the manufacturer was reached. These conditions produced spectra with high intensity particles generation (Figure 1b).

**Figure 2:**
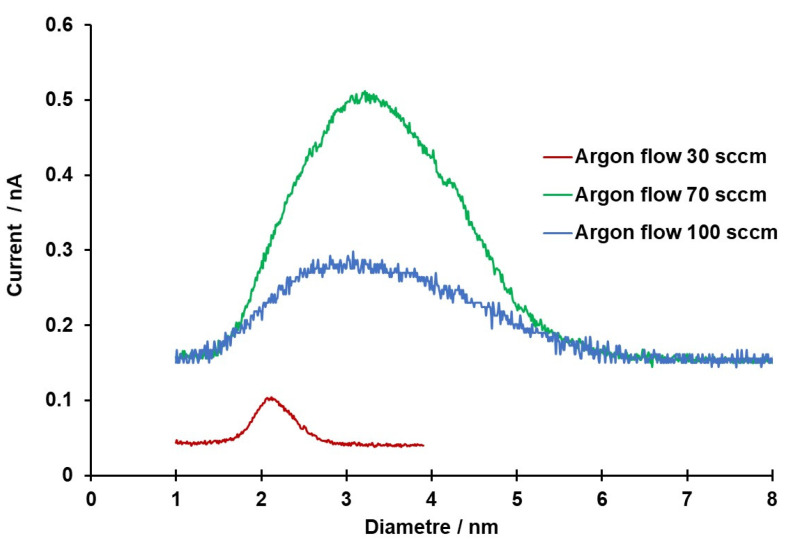
Comparison of spectra according to argon flow rate.

In addition to size pre-selection *via* MesoQ software, at fixed values of WD and temperature, size and size distribution of IONPs can be controlled changing the flow rate of the inert gas [42] in relation to the power and the throttle position (Table S3, Supplementary Information). To assess the effect of the inert gas (argon) flow rate at fixed WD and room temperature (20 °C), in this work, IONPs were generated using different flow rates and by applying enough power to achieve a stable plasma. The results depicted in Figure 2 show that IONPs production was not as high when slower argon flow rates were used (e.g., 30 sccm) and the production was at its maximum with the intermediate values of flow rate tested (70 sccm), when currents higher than 0.5 nA were recorded. The argon flow rate seems to affect not only the amount of particles generated, but also their size and distribution. In fact, at an argon flow rate of 30 sccm IONPs with size around 2 nm were produced, while flow rates of both 70 and 100 sccm produced particles with size between ~ 1–6 nm (Figure 2).

Therefore, by adjusting the sputtering conditions, the process can be repeated to obtain IONPs with the desired dimension and size distribution.

### 3.3. IONPs Characterization

**Figure 3:**
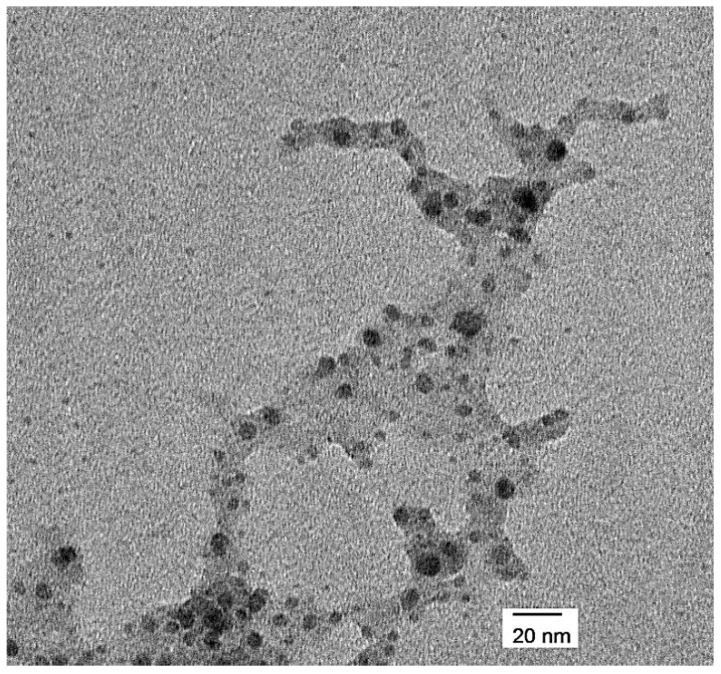
TEM micrographs of PEG encapsulated Fe_3_O_4_ NPs in RNAse free water.

IONPs produced from Fe_3_O_4_ targets at room temperature (20 °C) on silicon wafers coated with PEG, with an argon flow rate of around 70 sccm, were dispersed in 15 mL RNAse free water as described in the Materials and Methods section. As the amount of PEG spin-coated on the silicon wafer was estimated to be around 15.8 mg, the final concentration in the RNAse free water was calculated as 1.05 mg/mL. RNAse free water was selected as medium as the nanoparticles produced here will be investigated in the near future for delivery of gene therapy using RNA. A droplet (20 µL) of the IONPs suspension was then placed on an in-house, circular TEM grid (3 mm Ø), air dried and analyzed by TEM. The sample showed spherical IONPs (or aggregates) smaller than 20 nm, within a dry PEG matrix (Figure 3).

The TEM image confirms the presence of IONPs in the PEG solution eluted from the PEG coated silicon wafer after sputtering. Once the PEG IONPs solution was dried on the TEM grid for the measurements, the TEM image show that the majority of the particles are contained within the dry PEG layer. [22]. In addition, probably due to both further agglomeration during deposition on the wafer and the drying process of the PEG IONPs before measuring the sample, the nanoparticles seem to be present in aggregates and therefore show larger sizes than those observed during particles generation in the plasma chamber (Figure 2 and Figure 3). The TEM image also shows a small amount of IONPs outside the PEG layer.

**Figure 4:**
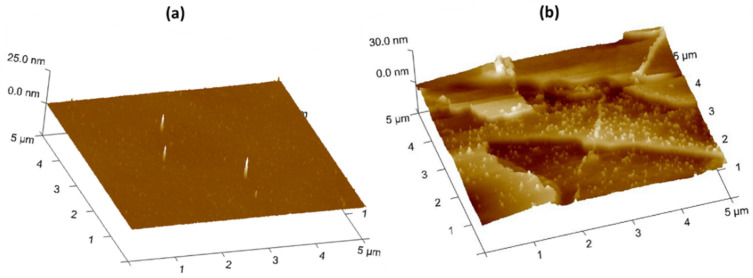
Bare Si wafer (**a**) and PEGylated IONPs dried on Si wafer (**b**).

To analyze the sample further, the suspended IONPs were blotted on a plain silicon wafer, air dried and characterized by AFM (Figure 4).

Comparison of the bare and coated substrates show topographical variation. The AFM images show an increased roughness due to the presence of the PEG layer (Figure 4b). The brighter spots on Figure 4b, might indicate presence of the IONPs within the PEG layer, confirming what was already seen in the TEM images.

**Figure 5:**
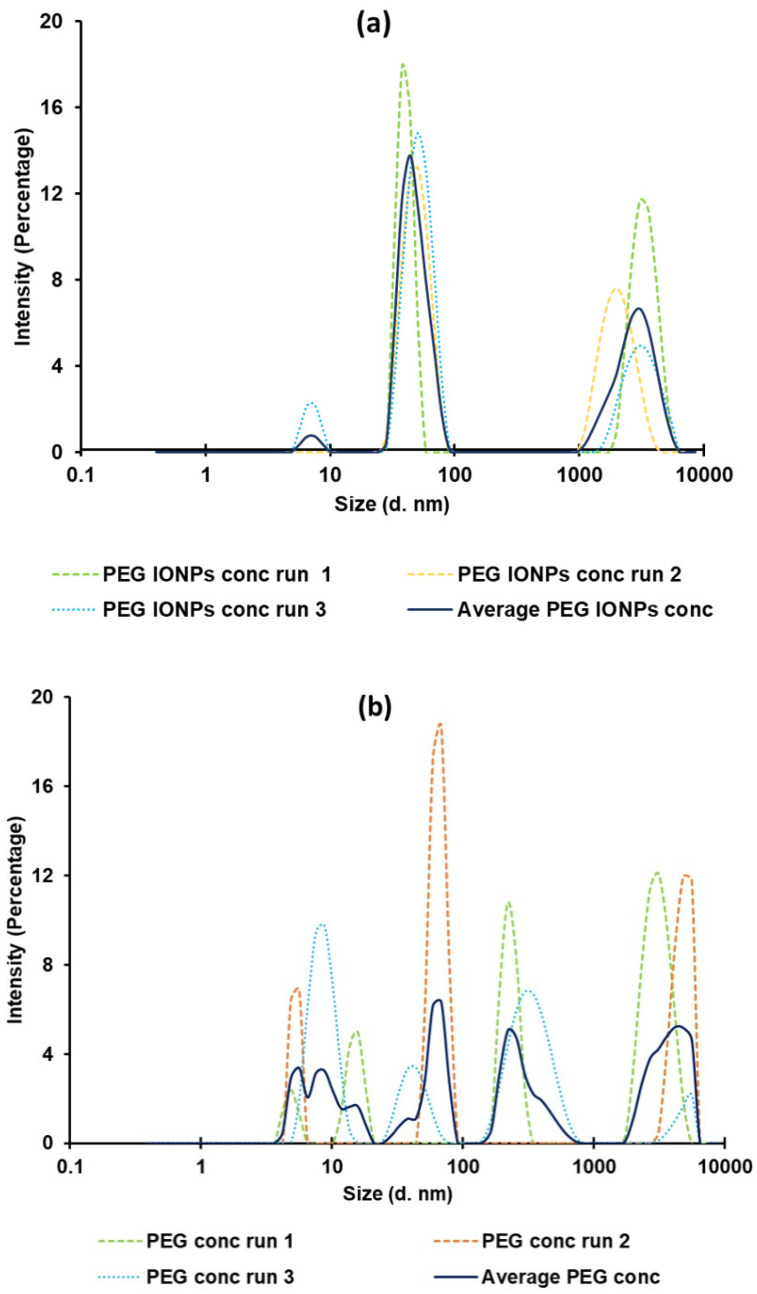
Dynamic light scattering of concentrated PEG IONPs solution (**a**) and concentrated PEG solution (**b**), as control. Averaged signal as well as individual runs are depicted.

In order to estimate the size and size distribution of the PEG-coated IONPs, the nanoparticles solution was concentrated 3 times and measured by DLS (Figure 5a). A solution containing only PEG, prepared as explained in the Methods section, was also concentrated 3 times and measured as control (Figure 5b) giving a final concentration of PEG in both solutions of around 3 mg/mL.

Figure shows that whereas we have distinct peaks in the PEG IONPs solution (Figure 5a) at 7 nm (bare IONPs), 47 nm (IONPs coated with PEG) and over 2860 nm (aggregation of PEG) with a polydispersity index (PDI) of 0.707 ± 0.098, the PEG solution (Figure 5b) shows a collection of peaks with a higher PDI (0.846 ± 0.160) demonstrating the absence of discrete nanoparticles. DLS measurements also indicate that the IONPs might be coated with around 40 nm of PEG. In the future, the PEG amount will be further optimized and most likely reduced, by changing the concentration of the PEG solution used for spin-coating the silicon wafer prior sputtering. In fact, a thinner layer of PEG around the IONPs should facilitate cell internalization, while maintaining the desired paramagnetic properties.

**Figure 6:**
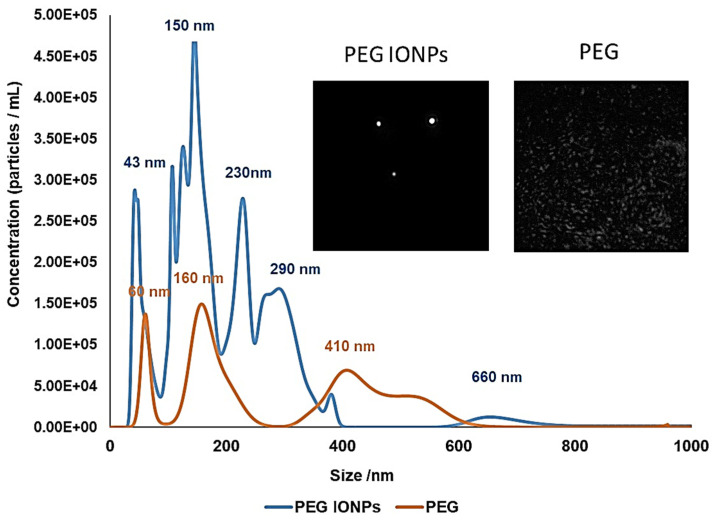
NTA measurements of concentrated PEG IONPs solution (blue) and concentrated PEG solution (orange), as control. Histograms are average of 3 measurements. Insets show screenshots of representative NTA video for both PEG IONPs and PEG only solutions.

In order to obtain further confirmation of the results observed by DLS, NanoSight Nanoparticles Tracking Analysis (NTA) of the two solutions (PEG IONPS and PEG only concentrated 3 times) was also performed. The NTA results are shown in Figure 6.

Both the graphs and the NTA videos (snapshots of the videos are reported as inset in the figure) showed a significant difference between the PEG IONPs and the PEG solutions. Whereas discrete particles were observed in the NTA videos of the PEG IONPs solution, (the bright spots on the left snapshot in the inset of Figure 6), no particles were seen in the videos of the PEG only sample. Nevertheless, a background signal was observed for both samples and particularly for the PEG only. This background signal is probably due to the high concentration of PEG (~3 mg/mL) present in both samples. Such high concentration of PEG is most likely affecting the NTA measurements and might be responsible for the difference in nanoparticles size observed between NTA and DLS analysis. Nonetheless, NTA as well as DLS, has further confirmed presence of free-standing and discrete IONPs dispersed in the PEG solution.

In conclusion, the characterization results have confirmed that free-standing PEG IONPs were successfully prepared and they were dispersible and stable in aqueous solution. In fact, the PEG IONPs samples produced here were stored in sealed containers (at −20 °C to prevent solution bacterial growth) and analyzed for weeks during the characterization without any noticeable deterioration of the samples.

## 4. Discussion

Iron Oxide nanoparticles with size < 20 nm (confirmed by TEM) were produced here at ambient temperature (20 °C). Whereas in this work the synthesis was performed at 20 °C to obtain particles ~ 10 nm, and therefore able to cross the cell membranes, if desired, larger particles (> 10 nm) can be obtained performing the sputtering at lower temperatures (e.g. −20 °C). The small particles produced here by our novel approach (IGC by the Mantis NanoGen Trio) has potential to be used for example to deliver gene therapy into target tissues. In addition, the novel production process used here allows us to obtain particles with narrow size distribution. This is desired as it enhances the predictability of the IONPs behavior in tissues as well as their excretion from the body after the release of the therapeutic agent.

Nanoparticles may be formed with spherical or cubic morphology using IGC by varying the flow rate of the inert gas in the source [30]. Furthermore, shape and size <15 nm can be controlled via precipitation of magnetite at low temperature in the presence of nitrogen gas [42]. The sputtering intensity at high or low power also affect the morphology, size and density of the produced IONPs and may affect the formation of the nanocarrier complex and attachment of biomolecules.

Direct production of metal nanoparticles coated with a biocompatible polymer such as PEG can be further modified with therapeutic agents (drugs) and antibodies to guide delivery to a specific cell receptor allowing particles to target specific cell types and successfully deliver therapeutic drugs [1].

The IGC production method used here has the added advantage of size pre-selection by regulating sputtering conditions and parameters. Furthermore, no additional purification steps are required after the IONPs are produced. If the process is prolonged, the IONPs form a thicker layer on the substrate increasing the yield of the material and influencing their magnetic behavior [43].

According to [44], nanoparticles are suitable for fabricating nanostructure materials, whose physical and chemical properties can be tailored. Nevertheless, IONPs size and size distribution cannot be easily controlled when they are produced by chemical methods [40]. Another major disadvantage of bulk solution nanoparticle synthesis, such as coprecipitation, is that the pH value of the reaction mixture has to be adjusted during synthesis and purification [45]. Furthermore, magnetic nanoparticles synthesis and physical methods such as gas phase deposition and electron beam lithography do not allow for the control of particle size [46]. The production method proposed here has shown that PEG - coated IONPs with a specific size and size distribution can be prepared by simple tuning the sputtering conditions.

In comparison to other organic polymers, PEG is biocompatible and commonly used to produce nanocarriers suitable for use in biological systems. The chemical nature of IONPs enables physical adsorption of the hydrophilic polymer on their surface, potentially reducing biotoxicity and accumulation in non-target tissues. Therefore, the free standing IONPs surrounded by PEG as a protective soluble coating obtained here, have the potential to be an efficient pay-loaded nanocarrier complex for transportation in biological systems and this is currently under investigation.

## 5. Conclusions

In this study, Fe_3_O_4_ targets were used to successfully sputter and deposit IONPs on PEG coated silicon wafer substrates using IGC technique by the Mantis NanoGen Trio system. Characterization of the resulting free-standing PEG coated IONPs has confirmed the suitability of the technique to produce PEG IONPs dispersed in an aqueous solution. The nanoparticles produced do not require further purification and are ready to be utilized; for example, for biomedical applications. Further work is currently investigating the chemistry to attach therapeutic agents and receptors for the guided and efficient delivery of RNA based drugs for enhanced gene therapy.

## Data Availability

Data is contained within the article or supplementary material.

## Supplementary Materials

The following are available online at https://www.mdpi.com/2306-5354/8/3/38/s1.
