# Supplementary material for: Surface Engineered Iron Oxide Nanoparticles Generated by Inert Gas Condensation for Biomedical Applications
Source: Bioengineering (Basel). 2021 Mar 15;8(3):38. doi: 10.3390/bioengineering8030038 (PMC8001625; doi:10.3390/bioengineering8030038)
Supplement: Supplementary file 1 [file bioengineering-08-00038-s001.pdf]

Surface Engineered Iron Oxide Nanoparticles Prepared by Inert Gas Condensation for Biomedical Applications

Aver Hemben, Iva Chianella and Glenn John Thomas Leighton *

Surface Engineering and Precision Institute, Cranfield University, Bedfordshire, UK;

a.hemben@cranfield.ac.uk; i.chianella.1998@cranfield.ac.uk; g.j.t.leighton@cranfield.ac.uk

Supporting Information

*Correspondence: g.j.t.leighton@cranfield.ac.uk

Scheme S1

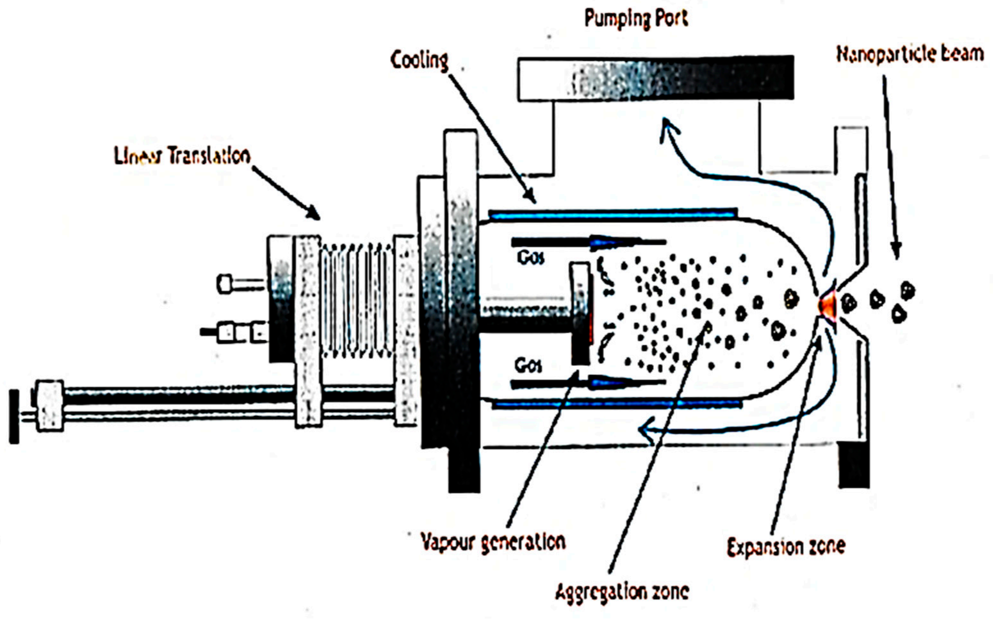

Scheme S1: Production of IONPs by inert gas condensation [33]

Figure S1.

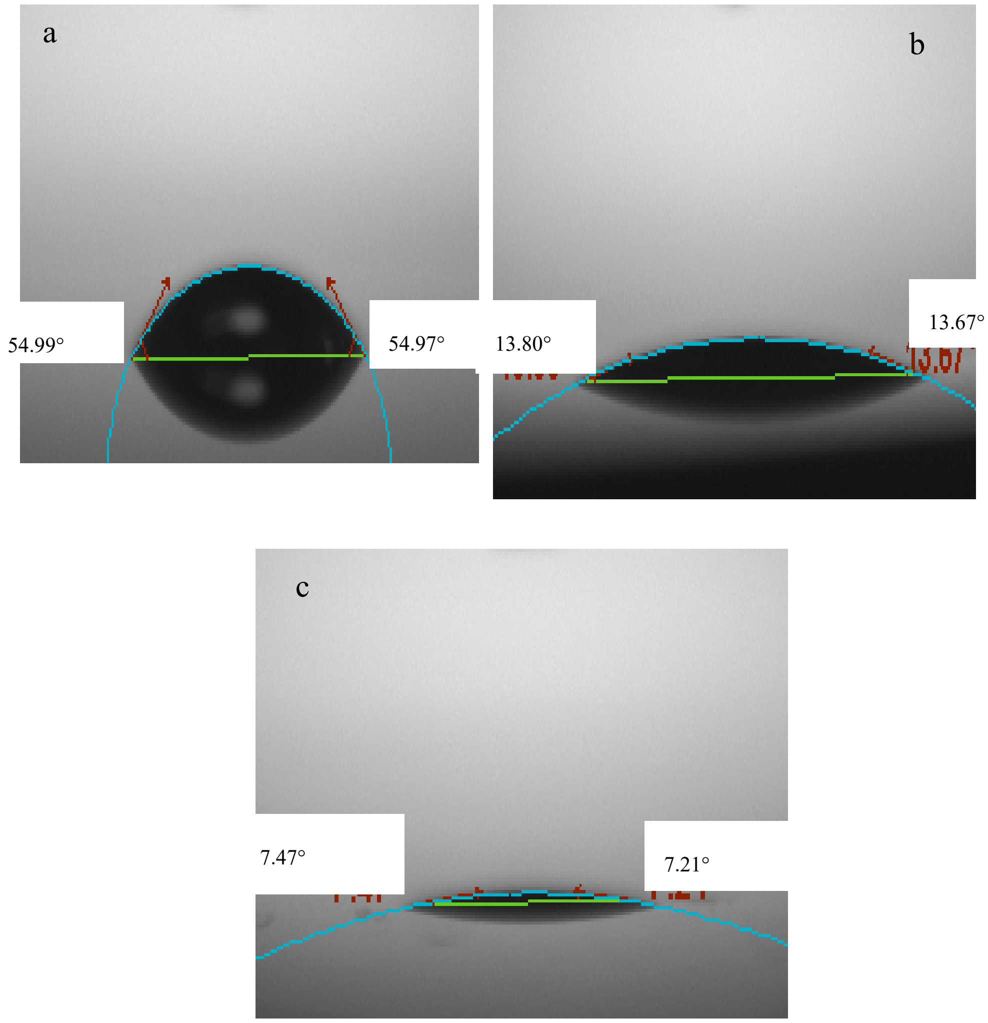

Figure S1: Contact angle measurement bare silicon wafer (a) PEG-coated wafer (b) IONPs deposited on PEG coated wafer (c).

Figure S2.

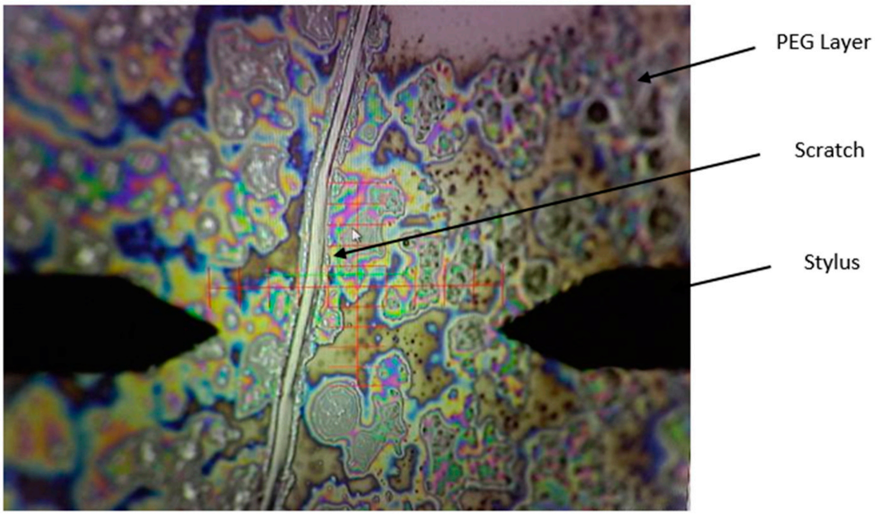

Figures S2. Scratched PEG-coated silicon wafer

Table S1

Table S1: Conditions tested to produce IONPs using the Mantis NanoGene Trio system.

Main chamber pressure (Torr)	Temperature (°c)	WD (cm)	Throttle position (%)
$2.84 \cdot 10^{-5}$	17	13.8	0
$7.36 \cdot 10^{-4}$	16	13.8	50
$1.32 \cdot 10^{-3}$	16	13.8	70

Table S2

Table S2: Mantis NanoGene Trio system parameters adjustment to achieve and maintain a plasma.

t_0 minutes		t_{30} minutes		t_{60} minutes		t_{90} minutes		t_{120} minutes	
Volts (V)	Amps (A)	Volts (V)	Amps (A)	Volts (V)	Amps (A)	Volts (V)	Amps (A)	Volts (V)	Amps (A)
454.0	0.110	455.0	0.110	456.0	0.109	438.0	0.109	458.0	0.103
459.7	0.109	404.5	0.108	456.0	0.107	468.0	0.107	468.0	0.107
460.0	0.109	465.4	0.106	467.0	0.106	461.1	0.107	463.0	0.109
468.0	0.107	466.5	0.107	468.0	0.107	467.0	0.101	466	0.107
458.5	0..114	472.1	0.114	488.0	0.110	488.0	0.100	487.0	0.110
492.1	0.100	490.0	0.100	492.3	0.100	491.2	0.100	493.5	0.100
531.9	0.038	531.6	0.030	529.0	0.030	528.3	0.030	524.2	0.031

Table S3

Table S3: Changes on the flow rate of argon in relation to the power and throttle position.

**Throttle position (%)**	**WD (cm)**	**Argon flow (sccm)**	**Average power (Watts)**
50	13.8	30	49.95
70	13.8	70	49.2
80	13.8	100	41.2
